# An Evaluation of Wetting and Adhesion of Three Bioceramic Root Canal Sealers to Intraradicular Human Dentin

**DOI:** 10.3390/ma11081286

**Published:** 2018-07-25

**Authors:** Jung-Hong Ha, Hyeon-Cheol Kim, Young Kyung Kim, Tae-Yub Kwon

**Affiliations:** 1 Department of Conservative Dentistry, School of Dentistry, Kyungpook National University, Daegu 41940, Korea; endoking@knu.ac.kr (J.-H.H.); wisekim@knu.ac.kr (Y.K.K.); 2 Department of Conservative Dentistry, School of Dentistry, Pusan National University, Yangsan 50612, Korea; golddent@pusan.ac.kr; 3 Department of Dental Biomaterials, School of Dentistry and Institute for Biomaterials Research & Development, Kyungpook National University, Daegu 41940, Korea

**Keywords:** adhesion, contact angle, intraradicular dentin, root canal sealer, wettability

## Abstract

Root canal sealers should have good wetting and adhesion with intraradicular dentin. This study evaluated the wetting and adhesion properties of three bioceramic root canal sealers on dentin using contact angle (CA) measurements and calculations based on the Owens–Wendt–Rabel–Kälble (OWRK) model and compared the properties with those of a resin sealer. Three bioceramic sealers (EndoSequence BC Sealer (BC); Endoseal MTA (EM); and MTA Fillapex (MF)) were tested, together with one epoxy resin-based sealer (AH Plus (AP)). Disc-shaped sealer specimens and human premolar teeth with flat and polished intraradicular dentin surfaces were prepared (*n* = 12). The CAs of two liquids (water and methylene iodide) were measured on the surfaces using the sessile drop method. The wetting and adhesion properties of the four sealers were calculated using the wetting envelope and isogram diagram, respectively. Group BC showed the best wettability among the four sealer groups. The best adhesion was achieved for group EM, followed by group BC, with a significant difference being present between the two groups (*p* < 0.05). The OWRK-based calculation indicated that the bioceramic BC and EM sealers showed superior wetting and adhesion properties to the AP sealers.

## 1. Introduction

A root canal sealer is indispensable during the root canal obturation procedure to accomplish a fluid-tight seal in the irregular root canal system. The use of a sealer with a thermoplastic core filling material (gutta-percha) is considered to be standard procedure in endodontic obturation [1]. An ideal sealer should offer appropriate physiochemical properties, such as a sufficient setting time, an excellent seal when set, insolubility against fluids, and biocompatibility [2,3,4]. Of the various types of root canal sealers that have been developed for use in clinical practice, bioceramic sealers have recently attracted interest mainly because of their excellent physicochemical and biological properties [5,6,7,8].

Adequate flow and wetting are important properties of root canal sealers during root canal obturation for adequate binding between the root canal walls and the main root filling material, helping achieve a fluid-tight and bacteria-proof seal [2,9]. Sealers should also have good adhesion with the dentin substrate, as well as with the core material to facilitate molecular attraction and allow either chemical adhesion or micromechanical attachment [1,10]. At present, there is no standard method used to measure the adhesion of a sealer to the root dentin [11]. Adhesive materials are frequently compared using bond strength and microleakage tests [1,11,12]. Although microleakage may be more important for endodontic applications than bond strength [12], a strong bond between the sealer and the root dentin is essential for maintaining the integrity of the interface during the post space preparation and during tooth flexure [11].

Contact angle (CA) is a practical indicator of the wetting behavior of a liquid material on a solid surface [13,14,15,16]. Ballal et al. [9] evaluated the wettability of root canal sealers on intraradicular dentin treated with different irrigants by measuring the CAs of sealer droplets placed on flat dentin specimens. However, when a sealer is not of low enough viscosity to be tested as a liquid, it is often difficult to measure its CA directly on dentin surfaces [17].

In this study, the wetting and adhesion properties of four commercial root canal sealers to intraradicular dentin surfaces were evaluated by testing the materials as solids and measuring the CAs of two probe liquids on their surfaces, as well as on dentin surfaces [14,18]. The surface energy parameters and their wetting and adhesion properties to intraradicular dentin were calculated from the observed CAs based on the Owens–Wendt–Rabel–Kälble (OWRK) two-component model [15,19,20].

## 2. Materials and Methods

### 2.1. Specimen Preparation

Three bioceramic sealers (EndoSequence BC Sealer (BC); Endoseal MTA (EM); and MTA Fillapex (MF)) and one epoxy resin-based sealer (AH Plus (AP)) were tested in this study. Their codes, manufacturers, compositions, and batch numbers are summarized in Table 1.

For the CA measurements, a total of 48 (*n* = 12 per material) disc-shaped sealer specimens (8 mm in diameter and 1 mm in thickness) were prepared. Cylindrical molds were placed on a Mylar polyester film over a glass slide. The materials were prepared according to their respective manufacturer’s instructions, filled into the mold, and covered with another film and then a glass slide. The assembly was clamped together and stored in a container at 37 °C with 100% relative humidity for 72 h. Twelve sound human premolar teeth, collected in accordance with the Institutional Review Board of Kyungpook National University Hospital (BMRI 74005-452) and with the informed consent of the patients, were embedded in epoxy resin and bisected longitudinally using a low-speed diamond under water cooling. Each root half was polished with 600-grit wet silicon carbide paper [9], irrigated with 5.25% sodium hypochlorite followed by final flush with 17% ethylenediamine tetraacetic acid solution for 1 min [21], rinsed with distilled water, and finally dried with paper points. An additional five specimens for each sealer group and the dentin were prepared as described above to check the surface roughness prior to the CA measurements because roughness changes over 0.1 μm alter the CA values [15,16].

### 2.2. Measurements

The average surface roughness (*R*_a_) of the sealers and the dentin was measured (three readings per specimen) using a calibrated profilometer (Surftest SV-400, Mitutoyo, Kawasaki, Japan). The stylus speed, cutoff, and range used were 0.1 mm/s, 0.25 mm, and 600 μm, respectively [16]. For the CA measurements, water and methylene iodide (MI) were used as the test liquids [15]. The CAs of the two liquids were measured on the sealer and the dentin surfaces using the sessile drop method on a CA goniometer (OCA 15 plus, DataPhysics, Filderstadt, Germany). For each measurement, the left and right CAs were averaged to obtain the final CA. All the CAs were measured in a temperature-controlled room at 23 ± 1 °C with relative humidity at 50 ± 5% [16,22].

### 2.3. Calculation of Wetting and Adhesion

The wettability of the sealers on the dentin surface was calculated based on the OWRK theory [15,19,20]. First, the Young–Dupré equation states the following [14,23]:

*W*_a_ = *σ*_l_(1 + cos*Θ*) (1)

where *W*_a_ is the thermodynamic work of adhesion between a liquid and a solid surface, *σ*_l_ is the surface tension of the liquid, and *Θ* is the contact angle [8,10]. According to the OWRK theory, *W*_a_ is described by the following equation [19,24]:

*W*_a_ = 2[(*σ*_l_^d^*σ*_s_^d^)^1/2^ + (*σ*_l_^p^*σ*_s_^p^)^1/2^] (2)

in which *σ*_s_ is the surface tension of the solid surface, and the superscripts d and p refer to the dispersive and polar components, respectively. The total surface tension *σ*_l_ is divided into two components as follows:

*σ*_l_ = *σ*_l_^d^ + *σ*_l_^p^. (3)

The surface energy parameters (in mN/m) of the two test liquids were as follows: *σ*: 72.8; *σ*^d^: 21.8; and *σ*^p^: 51.0 for water; and *σ*: 50.8 and *σ*^d^: 50.8 for MI [25].

When a liquid completely wets a surface (cos*Θ* = 1), the following equation is obtained [20,26,27]:

*σ*_l_^d^ + *σ*_l_^p^ = (*σ*_l_^d^*σ*_s_^d^)^1/2^ + (*σ*_l_^p^*σ*_s_^p^)^1/2^. (4)

The wetting parameter *R* is obtained from a simple geometric consideration as follows:

*R* = [(*σ*_l_^d^)^2^ + (*σ*_l_^p^)^2^]^1/2^. (5)

When (*R* cos*φ = σ*_l_^d^) and (*R* sin*φ = σ*_l_^p^) are incorporated into Equation 4, the following result is derived:

*R* cos*φ + R* sin*φ =* (*R* cos*φ σ*_s_^d^)^1/2^ + (*R* sin*φ σ*_s_^p^)^1/2^. (6)

Resolved to *R*, as a function of *φ*, the equation shows the value in the coordination system for complete wetting. When considering this function for *φ* in the range of 0–90°, *R*(*φ*) can be calculated to provide the wetting envelope for a certain surface energy as follows [20,26,27]:

*R*(*φ*) *=* {[(cos*φ σ*_s_^d^)^1/2^ + (sin*φ σ*_s_^p^)^1/2^]/(cos*φ* + sin*φ*)}^2^. (7)

The relation *R*(*φ*) applies only in the case of a complete wetting (CA = 0°). To expand the applicable angles, the parameter *R* is multiplied by the factor 2/(1 + cos*Θ*). In this study, the wetting envelopes for *Θ* = 10°, 20°, 30°, 40°, and 50° were entered.

The adhesion property of the sealers was also evaluated based on the OWRK theory [15,19,20]. From Equations 2 and 3, the following equation is obtained [19,24]:

*W*_a_ = [(*σ*_l_ + *σ*_l_^p^)*σ*_s_^d^]^1/2^ + (*σ*_l_^p^_s_^p^)^1/2^. (8)

For certain surfaces, *σ*_s_^p^ and *σ*_s_^d^ are constant; for a constant *W*_a_, a multi-parameter equation is calculated as follows:

*σ*_l_(*σ*_l_^p^) = {[*W*_a_/2 − (*σ*_s_^p^*σ*_l_^p^)^1/2^]^2^ + *σ*_s_^d^*σ*_l_^p^}/*σ*_s_^d^ (*σ*_l_^p^ ≥ 0). (9)

Then, isograms of *W*_a_ of liquids (sealers treated as solids in this study) in contact with a certain surface (dentin in this study) can be drawn. The polarity of the minimum *σ*_l_^p^_min_ can be calculated if the derivative 9 is set to 0, as follows [19,24]:

*σ*_l_^p^_min_ = [*W*_a_(*σ*_s_^p^)^1/2^/2(*σ*_s_^p^ + *σ*_s_^d^)]^2^. (10)

For each level of the *W*_a_, the value of the minimum polarity and corresponding surface tension can be determined, the combination of which will provide optimum adhesion. A straight line can then be plotted through these minima on the isogram. In this study, the perpendicular distance between each data point (*σ*_l_(*σ*_l_^p^)) of the sealers and the straight line on the isogram was calculated.

### 2.4. Statistical Analysis

For the surface energy parameter and isogram data, which did not meet the equal variance assumption (Levene’s test), the Kruskal–Wallis test was employed, followed by the Mann–Whitney post hoc test, with adjustment of significance levels using the Benjamini and Hochberg method for a multiple testing correction. The wetting angle data were analyzed using Fisher’s exact test. The significance level was set at 0.05.

## 3. Results

### 3.1. Surface Energy Parameters

**Figure 1:**
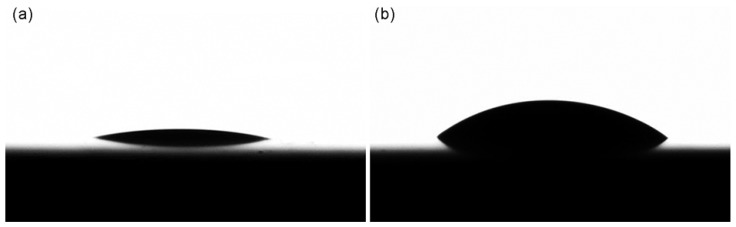
Drop topographies of water (**a**) and methylene iodide (**b**) formed on the dentin surfaces.

When the surface roughness of the sealer and the dentin specimens was checked prior to the CA measurements, the *R*_a_ values ranged from 0.14 ± 0.02 to 0.21 ± 0.10 μm. The surface energy parameters of the intraradicular dentin, which derived from the CA values (Figure 1), were as follows with the surface polarity being 45.37%: *σ*: 75.62; *σ*^d^: 41.29; and *σ*^p^: 34.32 (all in mN/m). Table 2 summarizes the surface energy parameters of the four sealers as also calculated from the CA data. Groups MF and AP showed significantly lower *σ* and *σ*^p^ values than did groups BC and EM, significant differences being present (*p* < 0.05). Group BC exhibited a significantly lower *σ*^d^ value (*p* < 0.05) than did the other three groups, in which there were no significant differences in the value (*p* > 0.05). On the other hand, group BC showed the highest *σ*^p^ value followed by group EM, a significant difference being present between the two groups (*p* < 0.05). There were no significant differences in any of the surface energy parameters between groups MF and AP (*p* > 0.05).

### 3.2. Wetting and Adhesion

**Figure 2:**
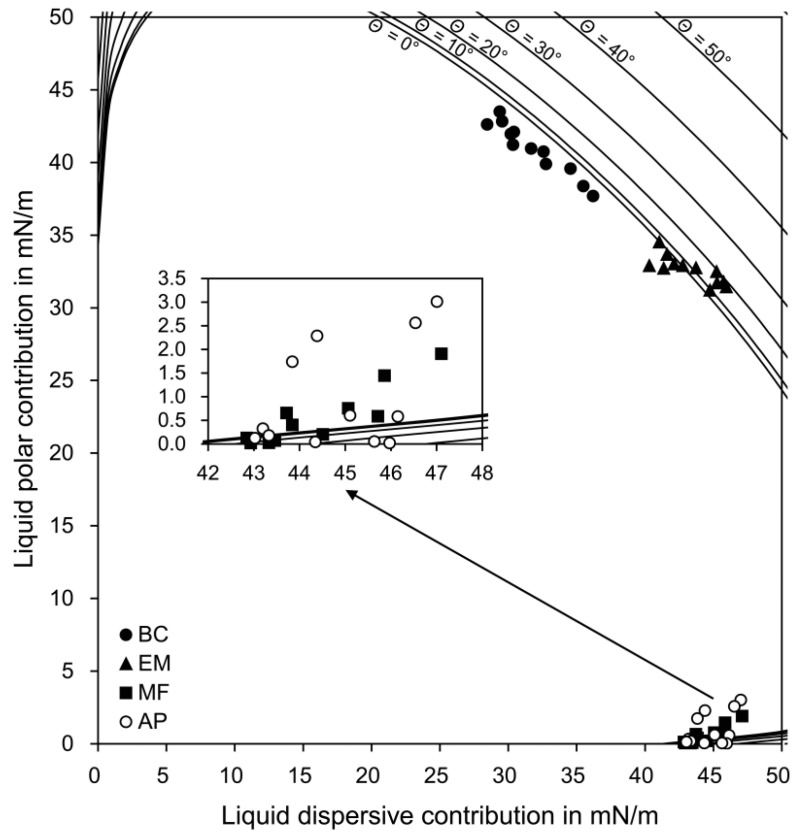
Wetting envelope of the dentin for 0–50°, showing each data point (*σ*^p^(*σ*^d^)) of the four sealers.

**Figure 3:**
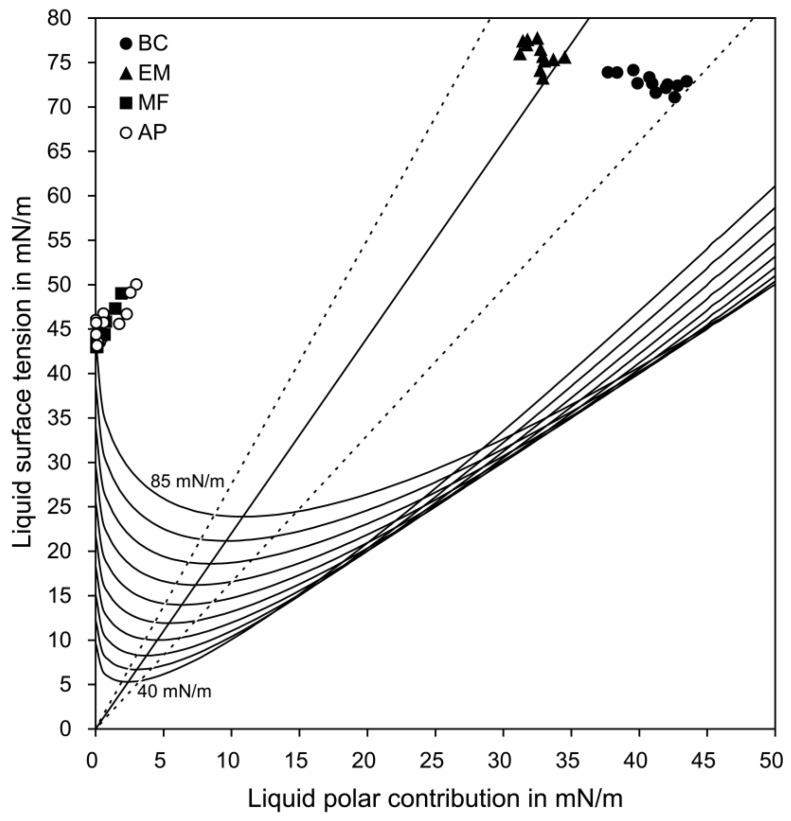
Diagram of the isograms corresponding to work of 40, 45, 50, 55, 60, 65, 70, 75, 80, and 85 mN/m, showing each data point (*σ*(*σ*^p^)) of the four sealers. The two dotted lines indicate a range of acceptable performance (± 25% of the optimum line in this study).

The calculation based on the OWRK theory (see Section 2.3) determined each data point of the four sealers on the wetting envelope (Figure 2) and the diagram of isograms (Figure 3). The wetting and adhesion properties of the sealers, which were calculated from the two figures, are summarized in Table 3. For the wetting envelope data, Fisher’s exact test indicated significant differences among the groups (*p* < 0.05). When considering the position of the *σ*^p^(*σ*^d^) values of the sealers on the wetting envelope, group BC showed the best wettability among the four sealer groups. The perpendicular distance between each data point (*σ*(*σ*^p^)) and the straight line on the isogram diagram indicate group EM as having the best adhesion, followed by group BC, with a significant difference being present between the two groups (*p* < 0.05). Groups MF and AP, with no significant difference (*p* > 0.05), had significantly poorer adhesion than did groups BC and EM (*p* < 0.05).

## 4. Discussion

Bioceramic root canal sealers have been reported to show excellent flow and appropriate film thickness, as well as favorable properties, including high calcium ion release, low dimensional change, proper radiopacity, and low solubility [2,5,6,7,8,28]. However, the wetting characteristics of bioceramic sealers have been rarely reported, even though they are crucial to adequate interaction between the root canal wall and sealers. The preliminary CA measurements indicated that none of the sealers selected, except for the BC sealer, formed a dome-shaped drop on the dentin surfaces, which is indispensable to determine the wetting and the CAs [9]. In this study, therefore, the surface energy parameters of the three bioceramic sealers were calculated by treating the materials as though they were solids and then measuring the CAs on the surfaces. The application of the wetting envelope and isogram diagram analyses (Figure 2 and Figure 3), both of which were based on the OWRK model [15,19,20], made it possible to predict the wetting and adhesion properties of the sealers on the dentin surfaces [19,20,24,26,27].

Based on the dispersive (*σ*^d^) and polar (*σ*^p^) components of the dentin surface energy, the wetting envelope of the dentin surface was drawn (Figure 2) [20,26,27]. The wettability of the sealers was defined by the size and shape of the envelope and the placement of the *σ*^d^ and *σ*^p^ components of the sealers deposited on the envelope [29]. The CAs of water (low) and MI (high) on the dentin surfaces (Figure 1) and the surface energy parameters indicate the hydrophilic characteristics of the intraradicular dentin surfaces [30,31]. The BC sealer showed the best wettability to the intraradicular dentin compared with the other sealers despite its high surface tension (*σ* value), probably because of its high hydrophilicity (Table 2). The wettability was slightly decreased in the EM sealer, which had a significantly higher *σ* value than the BC sealer. In groups MF and AP, all the data points still lay beneath the *Θ* = 40° curve on the wetting envelope (Table 3), indcating a relatively favorable wettability [20,26,27]. These results seem mainly attributable to the low *σ* values, despite hydrophobic characteristics.

Good wetting is a prerequisite to enhance adhesion between a root canal wall and a sealer because it enables penetration into the micro-irregularities [13]. However, good wetting does not necessarily indicate good adhesion [32]. When two dissimilar materials are in contact with each other, the extent of adhesive interactions (*W*_a_) depends on whether similar interactions can be formed between the two phases. The diagram of the isograms (Figure 3) was drawn based on the *σ*^p^ and *σ* values of the dentin surface energy [19,24]. The combination of the minimal *σ*^p^ value and corresponding *σ* value for each level of the *W*_a_ yields a straight line indicating optimal adhesion. Therefore, the distance between each data point (*σ*(*σ*^p^)) of the sealers and the straight line visually represent the adhesiveness of the sealers. In this study, the data points of group EM were the closest to the optimum straight line, indicating the best dentin adhesion. This finding was consistent with the observation that group EM had the most similar surface energy parameters with the dentin among the four sealers tested (Table 2). The MF and AP sealers exhibited poor adhesion to the dentin, probably because of their substantially low polar fraction (*σ*^p^ values) in comparison with dentin.

Overall, the bioceramic BC and EM sealers showed superior wetting and adhesion properties to the MF and AP sealers, suggesting that they would obturate irregular space and penetrate into dentinal tubules, thus enhancing the seal between the root canal surface and sealers. The bioceramic MF sealer is mainly composed of a combination of resins, silica, and MTA (Table 1). The material exhibited similar wetting and adhesion properties to the epoxy resin-based sealer AP (Table 3) due to their statistically similar surface energy parameters and hydrophobic characteristics (Table 2). This finding is comparable to the previous study by Assmann et al*.* [1], which found no significant difference in the dentin bond strength between MF and AP sealers.

In this study, the wetting and adhesion properties of four commercial root canal sealers to the intraradicular dentin were simply determined by measuring the CAs of the sealer and the dentin specimens, calculating their surface energy parameters, drawing the wetting envelope and isogram diagram, and then charting the surface energy parameters of the sealers on them. These procedures allow straightforward evaluation of the wetting and adhesion properties of formulation-modified sealer products. In particular, the surface tension and hydrophilicity/hydrophobicity of sealers should be optimized. Further studies are needed to analyze the dentin wettability and adhesion of root canal sealers depending on the type of root canal irrigants and irrigation methods. In this study, the wetting and adhesion properties of the sealers were not directly tested on the intraradicular dentin surfaces. If possible, therefore, such indirect evaluation of the sealer properties based on the CA measurements should be accompanied by direct tests for more comprehensive comparison.

## 5. Conclusions

This study evaluated the wetting and adhesion properties of three bioceramic sealers (BC, EM, and MF) on dentin using the CA measurements and calculations based on the OWRK model and compared the properties with those of a resin sealer (AP). The results indicated that the hydrophilic BC and EM sealers showed superior wetting and adhesion properties to the hydrophobic AP sealers. The hydrophobic MF and AP sealers had significantly poorer adhesion than did groups BC and EM.

## Figures and Tables

**Table 1:** Root canal sealers tested.

Brand Name (Code)	Manufacturer	Composition	Batch Number
EndoSequence BC Sealer (BC)	Brasseler USA, Savannah, GA, USA	Zirconium oxide, calcium silicates, calcium phosphate monobasic, calcium hydroxide, filler, and thickening agents	14004SP
Endoseal MTA (EM)	Maruchi, Wonju, Korea	Calcium silicates, calcium aluminates, calcium aluminoferrite, calcium sulfates, radiopacifier, and thickening agents	SEF670601
MTA Fillapex (MF)	Angelus, Londrina, Brazil	Paste A: salicylate resin, bismuth trioxide, fumed silica; Paste B: fumed silica, titanium dioxide, mineral trioxide aggregate, and base resin	35088
AH Plus (AP)	Dentsply DeTrey GmbH, Konstanz, Germany	Epoxide paste: diepoxide, calcium tungstate, zirconium oxide, aerosol, pigment; Amine paste: 1-adamantane amine, *N*,*N*′-dibenzyl-5-oxa-nonandiamin-1,9, TCD-diamine, calcium tongstate, zirconium oxide, aerosil, and silicon oil	1605000894

**Table 2:** Mean values (standard deviations) of surface energy parameters in mN/m for the four sealers (*n* = 12).

Groups	*σ* (Total Surface Tension)	*σ*^d^ (Dispersive Component)	*σ*^p^ (Polar Component)	Polarity (%)
BC	72.75 (0.93) ^a^	31.80 (2.54) ^a^	40.95 (1.80) ^a^	56.32 (3.00) ^a^
EM	75.91 (1.39) ^b^	43.32 (2.03) ^b^	32.59 (0.95) ^b^	42.96 (1.77) ^b^
MF	44.80 (1.94) ^c^	44.28 (1.38) ^b^	0.52 (0.61) ^c^	1.11 (1.25) ^c^
AP	45.84 (2.13) ^c^	44.88 (1.38) ^b^	0.96 (1.12) ^c^	2.01 (2.28) ^c^

Within each column, the same superscripted uppercase letters (a, b, and c) indicate statistically similar means (*p* > 0.05).

**Table 3:** Wetting and adhesion properties of the four sealers, derived from the wetting envelope and isogram diagram, respectively (*n* = 12).

Groups	Number of Each Data Point (*σ*^p^(*σ*^d^)) Lying beneath Each Curve on the Wetting Envelope					Perpendicular Distance between Each Data Point (*σ*(*σ*^p^)) and the Straight Line on the Isogram Diagram (mN/m)
	*Θ* = 0°	*Θ* = 10°	*Θ* = 20°	*Θ* = 30°	*Θ* = 40°	
BC	12	0	0	0	0	7.21 (1.92) ^a^
EM	5	2	5	0	0	1.74 (1.20) ^b^
MF	6	1	2	3	0	18.05 (0.33) ^c^
AP	7	1	1	1	2	18.08 (0.59) ^c^

For the wetting envelope data, Fisher’s exact test indicated significant differences among the groups (*p* < 0.05). For the isogram data (mean (standard deviation)), the same superscripted uppercase letters (a, b, and c) indicate statistically similar means (*p* > 0.05).

## References

[1] Assmann E., Scarparo R.K., Bottcher D.E., Grecca F.S. (2012). Dentin bond strength of two mineral trioxide aggregate-based and one epoxy resin-based sealers. J. Endod..

[2] (2012). Dentistry-Root Canal Sealing Materials.

[3] Cintra L.T.A., Benetti F., de Azevedo Queiroz Í.O., Ferreira L.L., Massunari L., Bueno C.R.E., de Oliveira S.H.P., Gomes-Filho J.E. (2017). Evaluation of the cytotoxicity and biocompatibility of new resin epoxy-based endodontic sealer containing calcium hydroxide. J. Endod..

[4] Rodríguez-Lozano F.J., García-Bernal D., Oñate-Sánchez R.E., Ortolani-Seltenerich P.S., Forner L., Moraleda J.M. (2017). Evaluation of cytocompatibility of calcium silicate-based endodontic sealers and their effects on the biological responses of mesenchymal dental stem cells. Int. Endod. J..

[5] Loushine B.A., Bryan T.E., Looney S.W., Gillen B.M., Loushine R.J., Weller R.N., Pashley D.H., Tay F.R. (2011). Setting properties and cytotoxicity evaluation of a premixed bioceramic root canal sealer. J. Endod..

[6] Candeiro G.T., Correia F.C., Duarte M.A., Ribeiro-Siqueira D.C., Gavini G. (2012). Evaluation of radiopacity, pH, release of calcium ions, and flow of a bioceramic root canal sealer. J. Endod..

[7] Candeiro G.T.M., Moura-Netto C., D'Almeida-Couto R.S., Azambuja-Júnior N., Marques M.M., Cai S., Gavini G. (2016). Cytotoxicity, genotoxicity and antibacterial effectiveness of a bioceramic endodontic sealer. Int. Endod. J..

[8] Lee J.K., Kwak S.W., Ha J.H., Lee W., Kim H.C. (2017). Physicochemical properties of epoxy resin-based and bioceramic-based root canal sealers. Bioinorg. Chem. Appl..

[9] Ballal N.V., Tweeny A., Khechen K., Prabhu K.N., Satyanarayan, Tay F.R. (2013). Wettability of root canal sealers on intraradicular dentine treated with different irrigating solutions. J. Dent..

[10] Dogan Buzoglu H., Calt S., Gümüsderelioglu M. (2007). Evaluation of the surface free energy on root canal dentine walls treated with chelating agents and NaOCl. Int. Endod. J..

[11] Al-Haddad A., Che Ab Aziz Z.A. (2016). Bioceramic-Based Root Canal Sealers: A Review. Int. J. Biomater..

[12] Schwartz R.S. (2006). Adhesive dentistry and endodontics. Part 2: bonding in the root canal system-the promise and the problems: A review. J. Endod..

[13] Grégoire G., Dabsie F., Dieng-Sarr F., Akon B., Sharrock P. (2011). Solvent composition of one-step self-etch adhesives and dentine wettability. J. Dent..

[14] Kim M.J., Kim Y.K., Kim K.H., Kwon T.Y. (2011). Shear bond strengths of various luting cements to zirconia ceramic: surface chemical aspects. J. Dent..

[15] Kwon S.M., Min B.K., Son J.S., Kim K.H., Kwon T.Y. (2016). Durability of resin bond strength to dental noble metal–ceramic alloys conditioned with novel mercapto silane-based primer systems. J. Adhes. Sci. Technol..

[16] Kim H.J., Bagheri R., Kim Y.K., Son J.S., Kwon T.Y. (2017). Influence of curing mode on the surface energy and sorption/solubility of dental self-adhesive resin cements. Materials.

[17] Benetti P., Della Bona A., Kelly J.R. (2010). Evaluation of thermal compatibility between core and veneer dental ceramics using shear bond strength test and contact angle measurement. Dent Mater..

[18] Kim I.H., Kim K.H., Son J.S., Kwon T.Y. (2017). Surface roughness effect on the solid equilibrium contact angle. J. Nanosci. Nanotechnol..

[19] Owens D.K., Wendt R.C. (1969). Estimation of the surface free energy of polymers. J. Appl. Polym. Sci..

[20] Vivet L., Joudrier A.L., Bouttemy M., Vigneron J., Tan K.L., Morelle J.M., Etcheberry A., Chalumeau L. (2013). Wettability and XPS analyses of nickel–phosphorus surfaces after plasma treatment: An efficient approach for surface qualification in mechatronic processes. Appl. Surf. Sci..

[21] Zehnder M. (2006). Root canal irrigants. J. Endod..

[22] Takimoto M., Ishii R., Iino M., Shimizu Y., Tsujimoto A., Takamizawa T., Ando S., Miyazaki M. (2012). Influence of temporary cement contamination on the surface free energy and dentine bond strength of self-adhesive cements. J Dent..

[23] Kim Y.K., Son J.S., Kim K.H., Kwon T.Y. (2013). Influence of surface energy parameters of dental self-adhesive resin cements on bond strength to dentin. J. Adhes. Sci. Technol..

[24] Waters M.G., Williams D.W., Jagger R.G., Lewis M.A. (1997). Adherence of Candida albicans to experimental denture soft lining materials. J. Prosthet. Dent..

[25] Ström G., Fredriksson M., Stenius P. (1987). Contact angles, work of adhesion, and interfacial tensions at a dissolving hydrocarbon surface. J. Colloid Interface Sci..

[26] Anderson L.J., Easton C.D., Jacob M.V. (2013). Compatibility of plasma-deposited linalyl acetate thin films with organic electronic device fabrication techniques. J. Mater. Sci..

[27] Ahmad J., Bazaka K., Oelgemöller M., Jacob M.V. (2014). Wetting, solubility and chemical characteristics of plasma-polymerized 1-isopropyl-4-methyl-1,4-cyclohexadiene thin films. Coatings.

[28] Zhou H.M., Shen Y., Zheng W., Li L., Zheng Y.F., Haapasalo M. (2013). Physical properties of 5 root canal sealers. J. Endod..

[29] Voigt M.M., Mackenzie R.C.I., King S.P., Yau C.P., Atienzar P., Dane J., Keivanidis P.E., Zadrazil I., Bradley D.D.C., Nelson J. (2012). Gravure printing inverted organic solar cells: The influence of ink properties on film quality and device performance. Sol. Energy Mater. Sol. Cells.

[30] Breschi L., Mazzoni A., Dorigo E.D.S., Ferrari M. (2009). Adhesion to intraradicular dentin: A review. J. Adhes. Sci. Technol..

[31] Kim Y.K., Min B.K., Son J.S., Kim K.H., Kwon T.Y. (2014). Influence of different drying methods on microtensile bond strength of self-adhesive resin cements to dentin. Acta Odontol. Scand..

[32] Samuel B., Zhao H., Law K.Y. (2011). Study of wetting and adhesion interactions between water and various polymer and superhydrophobic surfaces. J. Phys. Chem. C.

